# Long-term nutrition therapy leads to survival benefit in head and neck cancer patients receiving targeted or immunotherapy. A retrospective cohort study with real-world data

**DOI:** 10.3389/fonc.2025.1667150

**Published:** 2025-11-03

**Authors:** Barbara Belak, Erzsebet Palfi, Andrea Molnar, Celia Blasszauer, Daniel Reibl, Jozsef Lövey

**Affiliations:** ^1^ Doctoral School of Health Sciences, Semmelweis University, Budapest, Hungary; ^2^ Faculty of Health Sciences, Department of Dietetics and Nutrition Sciences, Semmelweis University, Budapest, Hungary; ^3^ MedicalScan Ltd., Budapest, Hungary; ^4^ Department of Oncology, Radiumhospital, Oslo University Hospital Comprehensive Cancer Centre, Oslo, Norway

**Keywords:** head and neck neoplasms, nutritional support, immunotherapy, molecular targeted therapy, survival

## Abstract

**Background:**

Nutritional status is one of the most important prognostic factors of survival in patients with recurrent or metastatic head and neck cancer (R/M HNC), and it can be positively influenced by medical nutrition therapy (MNT). To evaluate the impact of MNT, we collected Real-World Data on its use in R/M HNC patients receiving molecular targeted therapy (TT) or immunotherapy (IT) and examined the correlation between survival and duration of MNT.

**Methods:**

This retrospective, analytical, cohort study utilized data extracted from the electronic health records of the Hungarian National Health Insurance Fund Management. In total, data from 1,660 HNC patients treated between 2018 and 2023 were used. Statistical analysis was conducted using the Kaplan-Meier method, log-rank test, and Cox regression analysis. Survival was assessed over a follow-up period of 730 days.

**Results:**

During the study period, 993 of 1,660 patients (55.9%) aged over 18 received molecular TT, and 667 (40.1%) patients received IT. Patients were categorized into three groups based on whether they received MNT during treatment and the duration of MNT. When comparing these groups, we found that patients who received MNT for more than 6 months had better survival in both the targeted therapy group (HR = 0.49, 95% CI = 0.36–0.69, p<0.001) and the immunotherapy group (HR = 0.4, 95% CI = 0.22–0.72, p=0.002).

**Conclusions:**

Our study demonstrates a positive correlation between long-term use of MNT — defined as treatment lasting more than six months — and overall survival (OS) in patients with R/M HNC receiving TT or IT. These findings underscore the importance of early identification of inadequate nutritional status, as well as the timely initiation and sustained application of MNT. The main limitation of the research is that it is based on retrospective data and from only one country. Results may vary in other countries due to differences in treatment protocols and the composition of nutritional formulas.

## 1 Introduction

In recent years, numerous publications have highlighted several changes in the epidemiology and treatment of head and neck cancer (HNC) (1–7). HNC emerged as the 7th most common cancer globally, with rising incidence and mortality rates (1). Treatment modalities now include surgery, radiotherapy (RT), chemotherapy, targeted therapy (TT), and immunotherapy (IT) (4–8). Increased mortality in HCN patients can also be attributed to treatment-related side effects and severe malnutrition, which can interfere with therapy (9, 10). Treatment strategies for HNC depend on factors such as tumor stage, recurrence, and risk of secondary malignancies (8). Early-stage (I–II) cases are often treated with RT or surgery, while advanced stages (III–IVB) require multidisciplinary approaches. Personalized nutritional therapy is applied even in unresectable, recurrent, or metastatic cases (8).

In Hungary, around 4,000 new cases of head and neck squamous cell carcinoma (HNSCC) are diagnosed annually, with high mortality (11). Treatment usually follows European Society for Medical Oncology (ESMO) protocols, though access is sometimes limited by reimbursement policies. Poor nutritional status and comorbidities often hinder therapy, contributing to elevated mortality (12).

Given the high incidence, mortality, frequent relapses, poor prognosis, and decreasing average age of HNC patients, ongoing research into prevention, treatment, and supportive care remains essential. Our multidisciplinary Hungarian research team has focused on medical nutrition therapy (MNT) and previously found that patients receiving long-term MNT (≥7 months) had significantly improved overall survival (OS) (p < 0.0001) (13). In our current study, we narrowed the scope to patients undergoing TT and IT and redefined long-term MNT as therapy lasting more than 6 months to further explore its impact on survival outcomes.

## 2 Materials and methods

### 2.1 Ethics and patients

The study was performed in accordance with the Declaration of Helsinki. The ethical approval number is 276/2024.

This retrospective, nationwide, longitudinal study was conducted using data from the Hungarian National Health Insurance Fund (NHIF). The NHIF is a comprehensive national insurance system that provides coverage for nearly the entire Hungarian population. Its database includes patient identifiers, records of reimbursed prescriptions, a broad spectrum of medical interventions and healthcare events, as well as ICD-10 (International Statistical Classification of Diseases, 10th revision) codes for all inpatient and outpatient visits nationwide. In addition, pieces of demographic information such as age, sex, and dates of birth or death are recorded. For this study, we accessed specific NHIF sub-databases, including disease identification (ICD-10), diagnostics (Diagnosis-Related Groups, DRGs), and medical interventions (International Classification of Health Interventions, ICHI).

**Figure 1:**
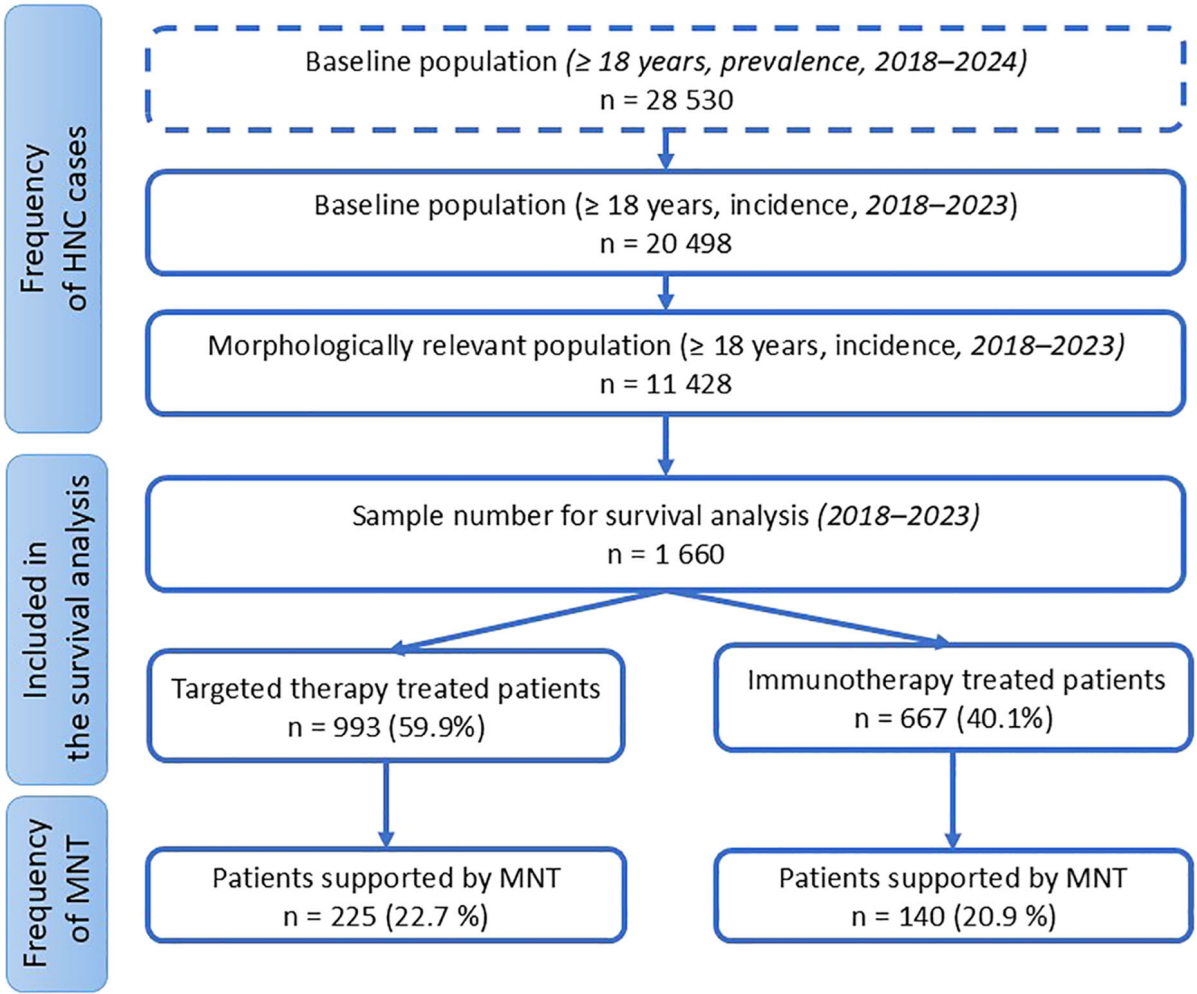
Patient numbers for subgroup analyses.

The total number of patients and subgroup distributions are shown in **Figure 1**, while the distribution of International Classification of Diseases (ICD) codes based on prevalence data is presented in **Supplementary Table 1**. The demographic characteristics of the study population based on incidence data are as follows: 14,146 males (69.0%) and 6,352 females (31.0%). By age group, 10,258 patients (50.0%) were aged 18–65 years, and 10,240 patients (50.0%) were over 65 years.

The term “medical nutrition therapy” (MNT) was defined as nutrition therapy interventions prescribed by a physician, specifically oral nutritional supplements (ONS) or enteral tube feeding (ETF). The research focused on ONS and ETF due to their considerably higher long-term usage compared to parenteral nutrition in Hungary. To estimate the frequency of HNC, we applied three restrictive inclusion criteria: ICD code, the study period, and adult age. From the ICD system, we included patients with codes C00–C14 and C30–C32, who had presented at least four times in inpatient or outpatient care (thereby excluding presumptive but unconfirmed cases). The analysis period spanned seven years (2018–2024) to assess prevalence, with six years (excluding the first year) used to identify incident cases. Patients under 18 years of age were excluded. Regarding oncological treatment, we specifically identified patients who received molecular TT or IT. In Hungary, the first-line treatment of R/M HNC is pembrolizumab monotherapy or in combination with platinum and 5-fluorouracil (5FU) in programmed death-ligand 1 (PD-L1) positive cases. The EXTREME or TPEx protocol is used in cases not amenable to IT (14–16).

Data on the use of MNT was retrieved from the prescribing data for foods for special medical purposes administered either orally (ONS) or via enteral feeding tubes (using enteral tube formula). In the first part of the survival analysis, we examined the relationship between MNT and survival in the two main groups of patients receiving anti-cancer treatment: TT or IT. For the second survival analysis, patients were categorized into three groups based on the duration of MNT: no or short-term MNT (1–3 months), medium-term MNT (4–6 months), and long-term MNT (>6 months of continuous therapy). These duration categories were based on standard follow-up intervals in the HNHIFM database (typically every 3 months) and on the national prescribing regulations, which allow physicians in Hungary to prescribe ONS and tube-feeding formulas for up to three months at a time.

Patients receiving MNT for only 1–3 months were classified as having no or short-term therapy, as our previous research indicated that short-term nutritional intervention does not significantly improve long-term survival (16). We hypothesize that three months is insufficient to achieve meaningful improvement in nutritional status, especially in patients undergoing active oncologic treatment. Survival was followed for a 730-day period.

### 2.2 Statistical analysis

All data extraction and statistical analyses were performed using “RStudio”. The available dataset included demographic data, treatment details, and data on MNT. Derived variables included frequencies of treatments and MNT use, as well as survival probability. A p-value of 0.05 is considered statistically significant for all tests, including the log-rank test. The Kaplan–Meier method was used to estimate survival probabilities. Log-rank tests were applied to compare survival distributions between groups. A Cox regression model was used to evaluate the association between the survival time and predictor variables. For the primary analysis, the control group consisted of patients who did not receive MNT or received it for ≤3 months, and the intervention group included those supported by MNT for ≥ 4 months. In the subgroup analysis, patients who received MNT were further divided into two subgroups based on the duration of nutritional therapy: medium-term MNT (4–6 months) and long-term MNT (> 6 months).

## 3 Results

### 3.1 Frequency of anti-cancer treatment

Among the HNC population, 14.5% (n = 1,660) of patients within the morphologically relevant subgroup (n = 11,428) received TT or IT in Hungary between 2018 and 2023 (**Figure 1**). For the survival analysis, data from these 1,660 HNC patients were included. Of them, 59.9% (n = 993) received TT, while 40.1% (n = 667) received IT (**Figure 1**).

### 3.2 Frequency of MNT

The proportion of patients receiving MNT was 22.7% (n = 225) among those treated with TT and 20.9% (n = 140) among those receiving IT (**Figure 1**).

### 3.3 Analyses of MNT and survival for patients who received TT or IT

**Figure 2:**
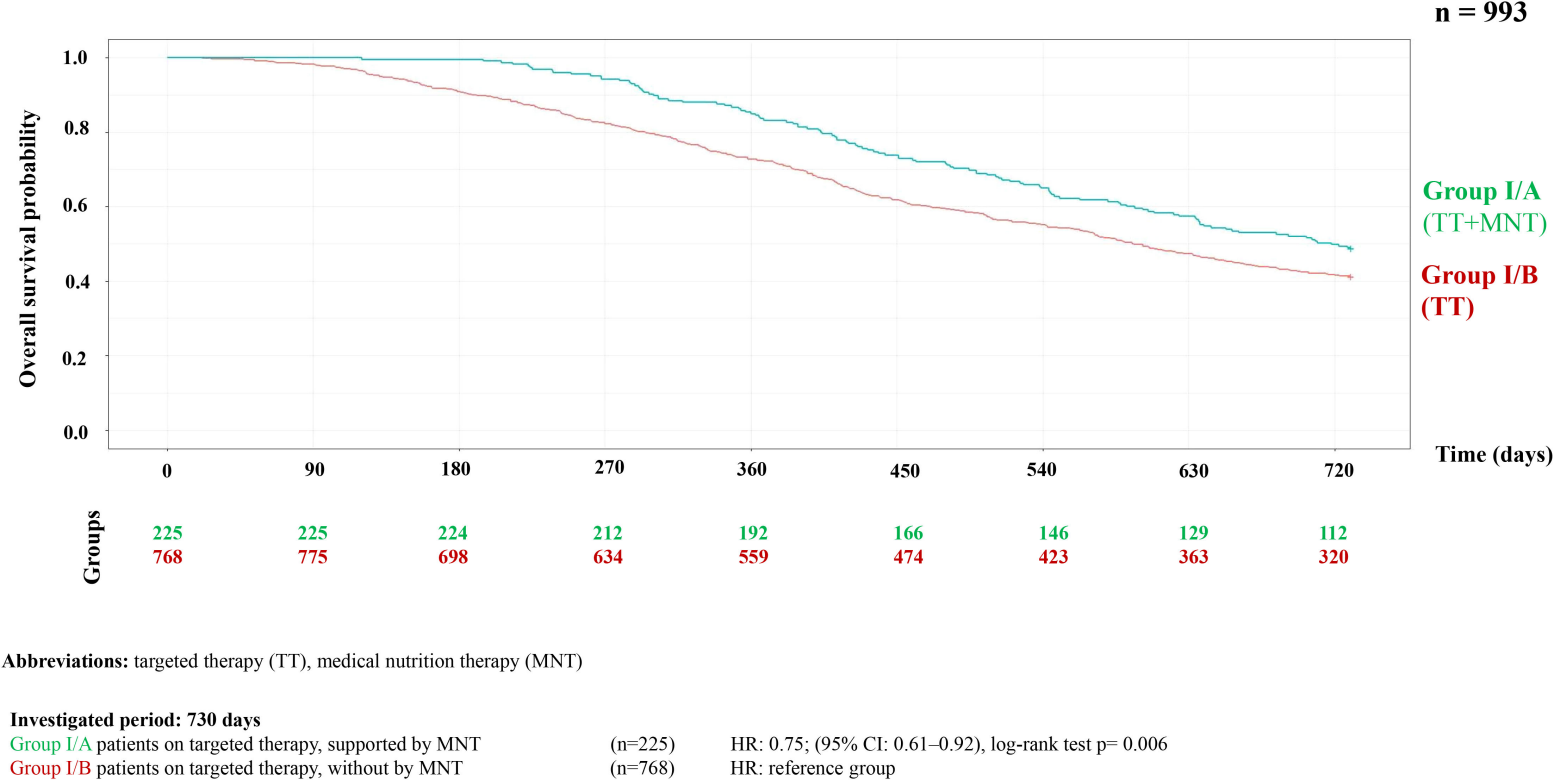
Kaplan-Meier curve of overall survival probability in HNC patients on targeted therapy with or without supported by MNT.

**Figure 3:**
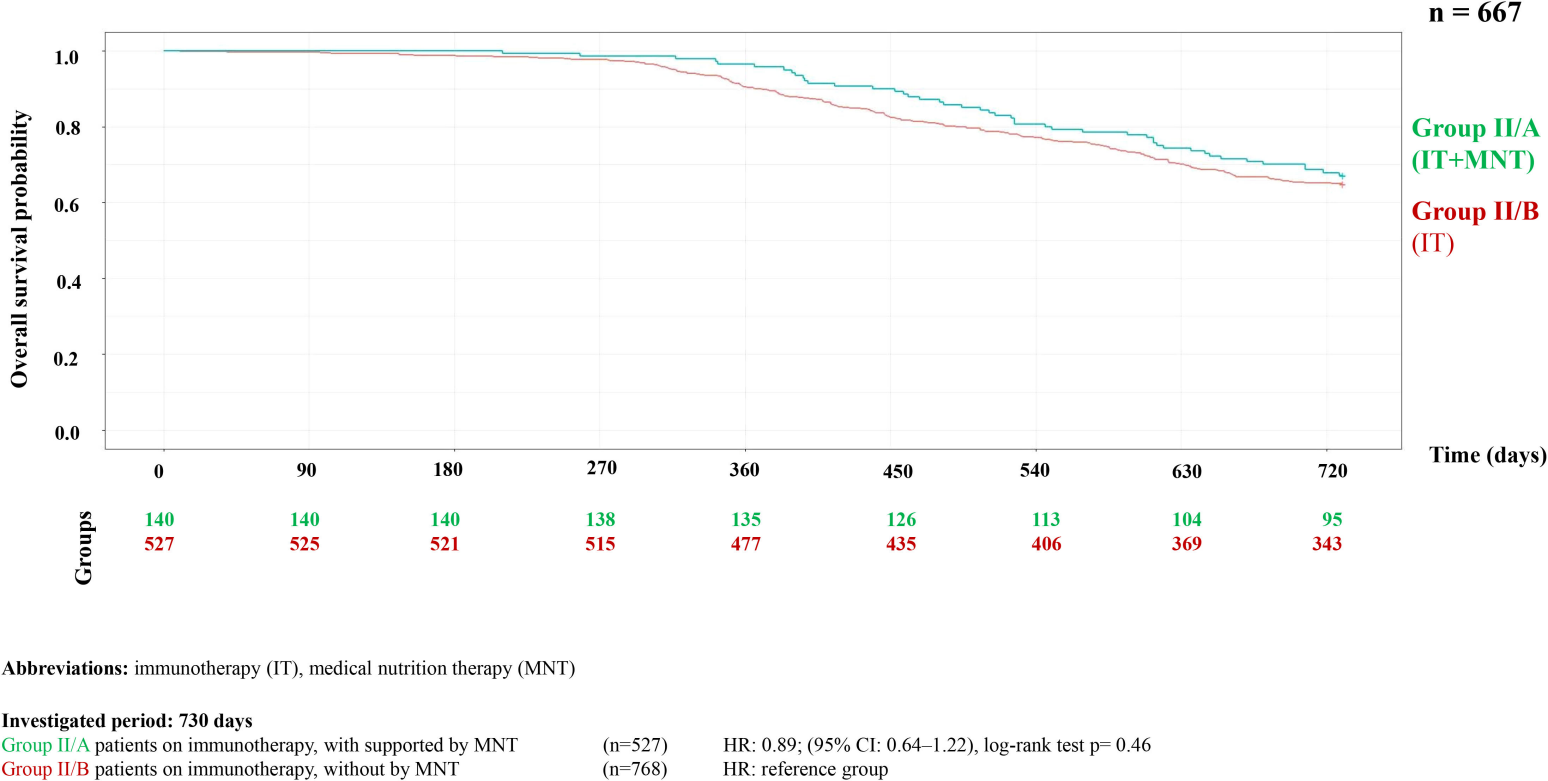
Kaplan-Meier curve of overall survival probability in HNC patients on immunotherapy with or without supported by MNT.

Analysis of MNT and survival among patients receiving TT or IT showed that MNT was associated with improved survival. This association was statistically significant in the targeted therapy group (p = 0.006), but not significant in the IT group (p<0.46) (**Figures 2**, **3**). **Table 1** presents the survival rates at the end of the first and second years, comparing patients with and without MNT. The data also indicate that patients receiving IT had better OS at both time points, and that MNT was associated with improved survival in both treatment groups.

**Table 1:** Survival rates in patients with and without medical nutritional therapy.

Type of therapy	Medical nutrition therapy	Number of patients	By the end of year 1		By the end of year 2	
			N	%	N	%
Targeted therapy						
	Intervention group	225	188	83.55%	110	48.88%
	Control group	768	558	72.65%	314	40.88%
Immunotherapy						
	Intervention group	140	135	96.42%	94	67.14%
	Control group	527	477	90.51%	342	64.89%

**Figure 4:**
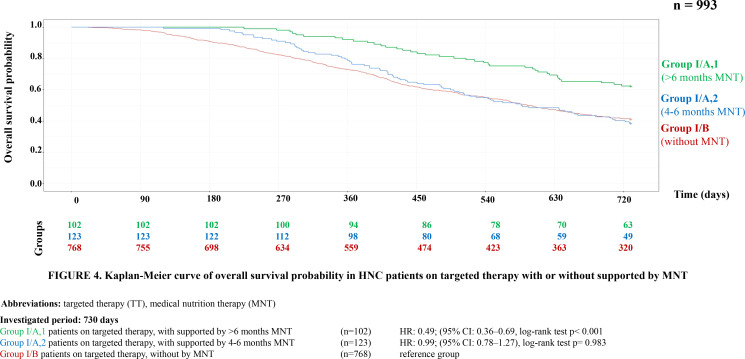
Kaplan-Meier curve of overall survival probability in HNC patients on targeted therapy with or without supported by MNT.

**Figure 5:**
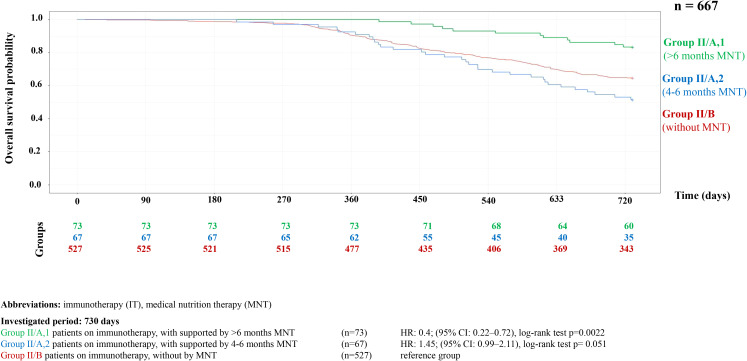
Kaplan-Meier curve of HNC overall survival probability in HNC patients on immunotherapy with or without supported by MNT.

Further survival analyses were conducted within each treatment group by categorizing patients into three subgroups based on the duration of MNT. The first group included patients who received no MNT or only for a short period (1–3 months), the second group included those who received medium-term support (4–6 months), and the third group received long-term support (>6 months). The survival probability was significantly higher in both the targeted therapy group (HR = 0.49, 95% CI = 0.36–0.69, p<0.001) and the immunotherapy group (HR = 0.4, 95% CI = 0.22–0.72, p = 0.002) (**Figures 4**, **5**).

## 4 Discussion

Concerning HNC patients, the number of long-term survivors is expected to increase in the coming years. This trend is driven by both shifts in patient demographics —from an older population with a history of smoking and alcohol use to a younger population in better general health — and the expansion of treatment options, including molecular TTs and ITs.

Recent clinical trials have demonstrated that these advanced therapies can significantly improve both survival and quality of life compared to standard treatments alone (17, 18). Therefore, it is increasingly important to understand and address the long-term supportive care needs of this growing survivor population, particularly concerning nutritional status and quality of life.

Among the publications examining survival in patients with head and neck cancer (particularly over the past decade), relatively few have focused on the impact of nutritional status and nutrition therapy on survival outcomes. Müller-Richter et al. reviewed factors influencing OS in HNC patients beyond cancer stage and treatment efficacy, emphasizing the role of nutritional status (19). They identified several independent predictors of survival related to nutrition, including body mass index (BMI), weight loss, malnutrition, and sarcopenia (19). Additional factors that, alongside nutritional status, were associated with poorer outcomes included: ECOG performance status >1, lymphocyte count <700/μL, elevated neutrophil-to-lymphocyte ratio, and postoperative infections (19). In our study, we found a positive correlation between the duration of MNT and OS in HNC patients. Our results showed that long-term nutritional support (≥7 months) significantly increased survival, primarily in the context of chemoradiotherapy and surgery (13). With the growing use of molecular targeted therapies and immunotherapies, there is an urgent need for similar survival data in patients receiving these new treatment modalities.

Regarding TT in HNC patients, several publications on cetuximab have explored its associations with nutritional status, malnutrition, sarcopenia, nutritional therapy, and survival. Authors of this article emphasize that although their research was conducted on patients with recurrent or metastatic (R/M) disease, no research is currently available that specifically examines the impact of nutritional therapy on survival in this population. Consequently, the present findings cannot be directly compared with those of other similar studies. Therefore, we compiled findings from studies focusing on locally advanced HNC treated with TT. Only two studies to date have directly examined the relationship between these factors and OS, specifically focusing on pathologically low muscle mass. Huiskamp et al. found through multivariate analysis, that low skeletal muscle mass in patients with head and neck squamous cell carcinoma treated with cetuximab and RT was significantly associated with reduced OS (Log Rank χ² = 5.87; *p* = 0.02), although it was not predictive of dose-limiting toxicity (OR 0.83; 95% CI 0.27–2.56; *p* = 0.74) (20). Similarly, Willemsen et al. identified low fat-free mass index (FFMI) as an unfavorable prognostic factor for survival, toxicity, and treatment tolerance in patients with locally advanced HNSCC (LAHNSCC) receiving chemoradiotherapy (CRT) or bioradiotherapy (BRT) (21). While associations between nutritional therapy and OS in patients receiving targeted therapy have not yet been clearly established, some studies have reported correlations with other clinical outcomes. In patients receiving TT, associations between nutrition therapy and OS have not yet been elucidated, although correlations with other clinical outcomes have been reported. Kapała et al. found that structured nutritional care (including dietary counselling and early enteral nutrition) improved treatment compliance and reduced complications in patients with locally advanced HNC undergoing CRT or BRT (22). Additionally, Berg et al. reported no significant differences in weight loss or malnutrition between cetuximab and cisplatin groups, though cetuximab was associated with reduced enteral nutrition needs and better physical functioning at the end of treatment (23).

In patients with HNC, emerging research has begun to explore the associations between nutritional status, nutritional therapy, and survival outcomes in the context of IT. Matsumura et al. investigated nutritional markers in patients with recurrent/metastatic HNSCC treated with PD-1 inhibitors and identified the Prognostic Nutritional Index (PNI) as an independent predictor of OS through multivariate analysis (24). Similarly, Gallen et al. found correlations between PNI and survival, focusing on pre-treatment nutritional status. In a retrospective analysis of 99 stage IV HNSCC patients treated with PD-1 and/or cytotoxic T-lymphocyte-associated protein 4 (CTLA-4) inhibitors, they evaluated baseline PNI and BMI trends and found that higher baseline PNI was significantly associated with improved OS in both univariate and multivariate analyses (17). These findings suggest that poor pretreatment nutritional status negatively impacts post-IT outcomes (17). Although not specific to HNC, Tran et al. reported that cancer-associated cachexia impairs the efficacy of immune checkpoint inhibitor (ICI) therapy (25). They also recommended routine assessment of PNI at the initiation of ICI treatment in clinical practice, given its predictive value for OS (25). Lin et al. investigated the effects of immunonutrition on immune function and clinical outcomes in HNSCC patients, particularly those receiving PD-1 inhibitors. This retrospective study included 49 patients and compared specialized immune-enhancing nutrition to standard care. Immunonutrition was associated with increased CD4+ and CD8+ T cell counts, reduced infection rates, and shorter hospital stays, suggesting that immunonutrition may enhance immune response and clinical outcomes, supporting its integration into comprehensive cancer care (25). Finally, Pannunzio et al. also emphasized the importance of a personalized and multimodal approach in the treatment of patients with HNSCC, integrating locoregional strategies (surgery and RT) with systemic therapies (chemotherapy, IT, and TT) (18). They highlighted that malnutrition remains a significant clinical problem across all treatment modalities, adversely affecting survival outcomes, thereby underscoring the critical role of nutritional therapy.

Currently, there are no specific European Society for Clinical Nutrition and Metabolism (ESPEN) or ESMO guidelines dedicated exclusively to nutritional therapy in HNC patients (26–28). General recommendations also address the timing of malnutrition risk screening and repetition of such screenings. Regarding nutritional therapy, they only provide guidance on when to initiate the intervention, but do not specify the recommended duration of nutritional support (26, 27). A 2024 review by Fan et al. summarized the best available evidence for nutritional support in patients with nasopharyngeal carcinoma undergoing RT, emphasizing the importance of timing in initiating support. However, their recommendations focused primarily on when to start nutritional therapy, without guidance on its duration (29). At present, there is no recommendation in nutritional guidelines regarding the therapeutic duration of MNT.

In conclusion, the authors emphasize the following key points: Previous research by our group, based on a large real-world dataset (RWD) from the HNC population, showed that long-term MNT (≥7 months) support in antitumor therapy can be significantly associated with improved survival (p < 0.0001) (13). Our current findings further support this, as RWE shows a positive correlation between long-term MNT support and improved survival even with newer treatment modalities such as TT and IT. Notably, this correlation remained significant even when the definition of long-term MNT was reduced to six months. However, to validate these retrospective findings, RCTs are necessary. Such studies could pave the way for including evidence-based recommendations on the optimal duration of MNT in clinical guidelines.

These results highlight the crucial role of multidisciplinary care across the patient journey, including early identification of nutritional risk and timely, sustained MNT intervention. The responsibility lies with all involved healthcare professionals to integrate nutrition support as a core component of oncological care.

During data analysis, we observed a notably low frequency of MNT (22.7% and 20.9% in the two patient groups), despite focusing on individuals with advanced recurrent or metastatic disease. Based on these findings, our research group would like to emphasize several key considerations. Adherence to both oncologic treatment and MNT is essential for optimizing outcomes in patients with HNC, particularly in recurrent or metastatic cases. However, adherence is often limited due to treatment-related side effects (pain, mucositis, and nausea), feeding difficulties (dysphagia, fatigue), and socioeconomic barriers (e.g., financial hardship, limited access to transportation, insufficient social support). A multidisciplinary approach (including dietetic support), patient education, and technology-assisted follow-up can help improve adherence and support comprehensive care. Moreover, the impact of MNT-focused patient education delivered by dietitians could be further strengthened by complementing individual counseling with small-group cooking workshops tailored to the specific needs of patients with dysphagia.

Limitations of our research include its retrospective study design, which provides real-world data analysis of MNT in unselected HNC patients undergoing TT and IT in Hungary. The results may not be generalizable to other countries due to differences in treatment protocols or the composition of oral and enteral feeding formulas. The study is also subject to several potential biases. Selection bias may be present, as the sample may not be representative of the entire HNC population. Information bias is possible due to limitations in the database, which lacked detailed data on patients’ nutritional status and the results of malnutrition, sarcopenia, cachexia screenings, or body composition (lean mass or fat-free mass), as well as quality of life (QOL) values. The NHIF database is subject to several important limitations. It does not capture data on mutation status, population-level histology, or therapies administered within clinical trials, as such treatments are not reimbursed by the social security system. Furthermore, the database lacks anamnestic variables (e.g., smoking history), TNM-based tumor staging, documentation of adverse events, ECOG performance status, laboratory parameters, and biomarker testing. Confounding bias may have occurred due to unmeasured variables that could affect survival outcomes. Additionally, the literature review was limited to studies published in English and Hungarian.

## Data Availability

The original contributions presented in the study are included in the article/**Supplementary Material**. Further inquiries can be directed to the corresponding author.

## Glossary

BW: body weight
CI: confidence interval
CPS: combined positive score
CRT: chemoradiotherapy
CTLA-4: Cytotoxic T-lymphocyte associated protein 4
DRGs: Diagnosis-Related Groups
ETF: enteral tube feeding
ESMO: European Society for Medical Oncology
ESPEN: European Society for Clinical Nutrition and Metabolism
5FU: 5-fluorouracil
FFMI: fat-free mass index
GI: gastrointestinal
HNC: head and neck cancer
HNSCC: head and neck squamous cell carcinoma
HNHIFM: Hungarian National Health Insurance Fund Management
ICD: international classification of diseases
ICHI: International Classification of Health Interventions
ICI: immune checkpoint inhibitors
IT: immunotherapy
LA: locally advanced
MNT: medical nutrition therapy
ONS: oral nutritional supplement
OS: overall survival
HR: hazard ratio
PNI: prognostic nutritional index
PD-L1: programmed death-ligand 1
QOL: quality of life
RCT: randomized controlled trial
RWD: real-world data
RWE: real-world evidence
R/M: recurrent/metastatic
RT: radiotherapy
TT: targeted therapy
