# Supplementary material for: Long-term nutrition therapy leads to survival benefit in head and neck cancer patients receiving targeted or immunotherapy. A retrospective cohort study with real-world data
Source: Front Oncol. 2025 Nov 3;15:1667150. doi: 10.3389/fonc.2025.1667150 (PMC12620219; doi:10.3389/fonc.2025.1667150)
Supplement: Supplementary file 1 [file Table1.pdf]

Supplementary Material

Supplementary Table 1. Prevalence and Distribution of Head and Neck Cancers in Hungary Between 2018 and 2024

ICD Codes	International Classification of Diseases (ICD), Topographical Codes	2018	2019	2020	2021	2022	2023	Summary
**C00**	Lip	656	755	720	738	786	833	**3 202**
**C01**	Base of tongue	871	861	791	746	744	719	**2 489**
**C02**	Other and unspecified parts of the tongue	796	857	775	791	790	836	**2 639**
**C03**	Gum	193	177	165	195	194	214	**674**
**C04**	Floor of the mouth	575	540	470	477	483	476	**1 552**
**C05**	Palate	332	319	243	247	212	229	**921**
**C06**	Other and unspecified parts of the mouth	298	293	266	316	308	317	**1 093**
**C07**	Parotid gland	366	428	401	356	333	373	**1 309**
**C08**	Other and unspecified major salivary glands	139	186	145	152	184	188	**590**
**C09**	Tonsil	787	763	659	633	631	611	**2 051**
**C10**	Oropharynx	824	814	775	770	764	765	**2 684**
**C11**	Nasopharynx	324	331	282	279	282	258	**831**
**C12**	Pyriform sinus	126	111	91	92	69	56	**287**
**C13**	Hypopharynx	1 279	1 242	1 097	1 000	927	878	**3 430**
**C14**	Other and ill-defined sites in the lip, oral cavity, and pharynx	1 050	1 085	1 015	914	940	947	**3 195**
**C30**	Nasal cavity and middle ear	230	225	218	223	212	224	**843**
**C31**	Accessory sinuses	176	169	148	153	151	140	**557**
**C32**	Larynx	3 293	3 151	2 761	2 742	2 669	2 626	**7 827**
**Summary**		**10 638**	**10 632**	**9 582**	**9 364**	**9 318**	**9 303**	**28 530**
